# Rotations of macromolecules affect nonspecific biological responses to magnetic fields

**DOI:** 10.1038/s41598-018-31847-y

**Published:** 2018-09-10

**Authors:** Vladimir N. Binhi, Frank S. Prato

**Affiliations:** 1 Prokhorov General Physics Institute, Moscow, 119991 Russia; 2 Lawson Health Research Institute, Ontario, N6A 4V2 Canada

## Abstract

We have previously proposed that there are at least two initial molecular transduction mechanisms needed to explain specific and nonspecific biological effects of weak magnetic fields. For the specific effect associated with animal magnetic navigation, the radical pair mechanism is the leading hypothesis; it associates the specialised magnetic sense with the radical pairs located in the eye retina. In contrast to the magnetic sense, nonspecific effects occur through the interaction of magnetic fields with magnetic moments dispersed over the organism. However, it is unlikely that the radical pair mechanism can explain such nonspecific phenomena. In order to explain these, we further develop our physical model for the case of magnetic moments residing in rotating molecules. It is shown that, in some conditions, the precession of the magnetic moments that reside on rotating molecules can be slowed relative to the immediate biophysical structures. In terms of quantum mechanics this corresponds to the mixing of the quantum levels of magnetic moments. Hence this mechanism is called the Level Mixing Mechanism, or the LMM. The results obtained are magnetic field-dependences that are in good agreement with known experiments where biological effects arise in response to the reversal of the magnetic field vector.

## Introduction

A large number of different biological effects can be observed at weak magnetic fields (MFs) in the range 0.1–100 *μ* T, e.g.^1^. For migratory animals that have formed their specific magnetic sense in the course of evolution, the initial transduction mechanism is apparently associated with spin-correlated radical pairs in retina cryptochromes, or with the radical pair mechanism (RPM), e.g.^2,3^. In contrast, there is a so-called nonspecific response to magnetic fields that is characteristic of all organisms and manifests itself only occasionally, at random effective combinations of electromagnetic and biochemical/physiological conditions^4^. This nonspecific response differs from magnetoreception in navigating animals, and the related biophysical mechanism remains largely unknown.

We have shown that the effects of a hypomagnetic field (HMF) occupy a special place among the nonspecific effects due to their higher magnitude and reproducibility and their capability of providing more precise information on the origin of the MF effects^4^. We define HMF when both the static (*H*) and the rms value of the variable component (*h*) are much less than the geomagnetic field ${H}_{{\rm{g}}} \sim 50\,\mu {\rm{T}}$, and the resultant electric currents induced by *h* can be considered negligible.

Special terms have been introduced for those micro objects that react with MF at the beginning of a signal transduction path and thus can be referred to as the targets of the MF. These are (i) primary physical targets, i.e., magnetic moments, and (ii) molecular or biophysical MF targets, or sensors that carry the moments and can change depending on the state of the moments.

The existence of HMF effects can be illustrated in terms of quantum mechanics. The Zeeman sublevels of a magnetic moment will degenerate, or mix, when their width becomes comparable to the Zeeman splitting caused by the applied magnetic field. Then a critical MF exists that defines the MF magnitude below which changes should occur at the quantum level. In mathematical notation, the width of the sublevels is of the order of ℏ/*τ*, where *τ* is the thermal relaxation time, and ℏ is the reduced Plank constant, while the splitting is ℏ*γH*, where *γ* is the gyromagnetic ratio. Then the critical MF is of the order of 1/*γτ*.

A physical mechanism for HMF effects has been proposed^5,6^, which considers the dynamics of non-uniformly precessing magnetic moments in biophysical targets, or MF sensors, that are not specialised MF receptors. Magnetic effects occur as the consequence of a significant slowing of the precession of magnetic moments, or, in a quantum picture, the degeneracy, or mixing, of their quantum levels. Therefore, in what follows, the mechanism proposed is referred to as the “level mixing” mechanism (LMM). The mechanism predicts MF-dependences that agree with experiments.

In this article, we relate the theoretical predictions of the LMM to two features of experimental observations that cannot be explained by the RPM. The first one is that the reversal of the static MF vector (**H **→ −**H**) causes a biological effect, indicating that the *H*-dependence is asymmetric. The second feature is that the *H*-dependencies are not monotonic, i.e., as the *H* field is ramped up, peaks in the biological effects occur.

Asymmetric effects have been previously reported on gene expression in *A. thaliana*^7^ and on calpain activity in mollusk *L. stagnalis*^8^. An asymmetric response to MF reversal has been found in many migrating animals, e.g.^9,10^, where this ability is associated with a so-called polar magnetic compass sense.

Many observations^7,11–15^ demonstrate the response of a plant *Arabidopsis thaliana* to a weak MF, although at least one researcher has not been able to reproduce this effect^16^. Recent experiments^13^ show that a weak static MF affects the expression of some genes in *A. thaliana*, where a few well-resolved peaks are observed in MF-dependences. Multi-peak MF-dependences have been previously observed in *E. coli* cells^17^ and in the planaria *Dugesia tigrina*^18^.

It seems unlikely that the RPM could explain these results due to experimental^7–10,13^ and theoretical^4^ reasons (see Appendix). The RPM is not sensitive to rotations of the molecular environment that carries the radical pair, because the probability of its chemical reaction depends on the *relative* orientation of the two spins. The asymmetric and multi-peak magnetic response in *E. coli*^17^ has been well described by the interference mechanism^19^ extended to the case of molecular rotations^20^. The interference mechanism and LMM are cognate: both predict preferred angular positions of their objects in MFs with certain characteristics. In the present work, the LMM theory adapted for molecular rotations is used to explain the asymmetric response to the MF reversal and the multi-peak character of magnetic effects in organisms and of gene expression in *A. thaliana*. As concluded in^21^, light-dependent RPM is not the only relevant mechanism, and work on alternative mechanisms like^6^ should continue^22^.

**Figure 1:**
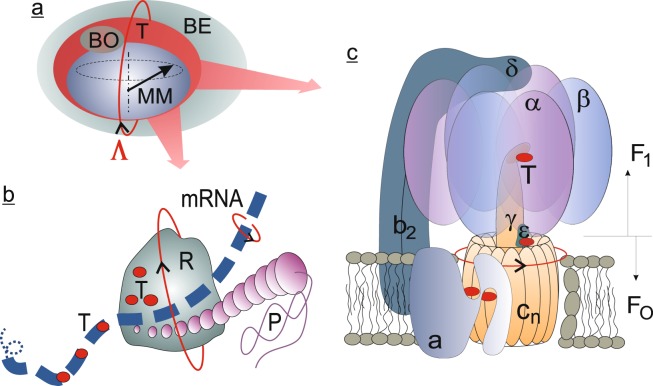
(**a**) A molecular MF target T (MF sensor) that rotates with angular velocity **Λ** together with the immediate biophysical environment BE. The MF sensor includes a precessing magnetic moment MM and an adjacent biophysical object BO, the state of which changes coherently with MM. (**b**) Possible positions of the MF sensors T (shown by small red circles) on a rotating mRNA molecule and/or inside oppositely rotating ribosome R during a protein P translation. (**c**) F_O_F_1_-ATPase electrochemical motor and its main subunits, based on data, e.g^50^. and references therein. The rotor that is composed of the c-ring and *γ*-stalk is shown in orange. The membrane and subunits a, b_2_, *δ*, three *α*, and three *β* serve as the stator. Possible positions of the MF sensors T are the places of the core ATPase events: ATP synthesis/hydrolysis and proton transportation.

Rotations are ubiquitous at the level of molecular processes, particularly in many of those related to DNA, RNA, and ATPase, Fig. 1. As previously suggested^17,23^, rotations of the molecules that carry the precessing magnetic moment can significantly affect the process in which the magnetic moment initiates subsequent biophysical events.

The aim of the present study was to show that the LMM, unlike the RPM, can explain the asymmetry of nonspecific magnetic response with regard to the MF vector reversal. This explanation posits an important and general question on the role of molecular rotations in magnetic biology.

Below we extend the LMM to the case where the molecular surrounding of magnetic moments rotates with a natural biological speed. The probability of secondary biophysical events in the MF sensors that reside on rotating molecules is estimated and *H*-dependences of this probability are calculated. Based on comparison with the experimental observations of others, it is concluded that rotating macromolecules are involved in nonspecific response to MF in organisms, and the molecular MF sensors reside on such macromolecules.

## Rotations of the Molecular MF Sensors

It is interesting to note, that reproducible and large nonspecific magnetic effects are observed in systems with pronounced processes involving gene expression: neurite outgrowth^24^, cephalic regeneration in planarians^25^, morphological changes during embryogenesis^26^, response to heat shock^27^, cell growth and gene expression^7^ in plants, the proliferation of human neuroblastoma cells^28^ and of mouse nerve stem cells^29^, and gravitropism in plants^30^. The combined action of MF and X-rays^31^ and of MF and heavy ions^32^ can be seen as the interference between DNA repair and nonspecific magnetic effects. There is also strong dependence of magnetic effects on the genetic modification of organisms^14,33–36^. Taken together this suggests that gene expression could be a prerequisite for MFs to elicit a nonspecific effect.

Transcription and translation are known to be accompanied by the rotation of macromolecules. For example, ribosome and its parts rotate in the process of translation^37^. Motion of RNA polymerase and helicase along the DNA helix is accompanied by relative rotation of the enzyme and DNA. Several examples of the macromolecular rotations in *E. coli* cells with speeds from a few to a few hundred rps have been reported^17^ which implies that there are rotations of DNA topoisomerase, DNA and RNA polymerases, F_*O*_ F_1_-ATPases, and the flagellar motor. Other contenders for rotating macromolecules include myosin rotation about an actin filament at 1.5–2.5 rps^38^ and rotation of microtubules induced by dynein at 1–4 rps^39^.

Thus, when modeling magnetic biological effects, it makes sense to take into account the rotations of the immediate environment of magnetic moments, i.e., the rotations of the MF sensor itself.

If an organism is experiencing a spatial shift along any axis in the laboratory frame, then shifted are all its elements including atoms and molecules together with their magnetic moments. It might seem that if the body rotated, all of its elements would rotate with it also. However, this is not the case with respect to elementary precessing angular momenta and their magnetic moments. Due to physical laws, rotational motion of the MF sensor body does not transmit the torque to the precessing angular momentum. For example, while holding a gyroscope, it is easy to move its body linearly, but it is difficult to roll out or slow down its rotor–this would require specific movements. Similarly, a magnetic moment precesses mostly independently of the molecular enclosure; although the moment’s thermal relaxation is due to the interaction with the enclosure.

If the angular velocity **Λ** of the rotating body, e.g., BO in Fig. 1a, is close to that of the magnetic moment precession *γ***H**, there will be a situation similar to the temporary slowing of the moment, in the rotating frame of the body, or a level mixing. A HMF effect arises, although the MF is not a HMF. This mechanism resembles a roulette wheel–the ball falls in the cell when the angular velocities of the ball and of the roulette wheel coincide. Similarly, HMF effect occurs when there is a coincidence of the angular velocities of the precession of the magnetic moment and of the sensor body.

In general, an organism may contain various types of sensors that rotate with several different speeds. In this case, when scanning the dc MF magnitude, the effect, like a HMF effect, will occur sequentially for sensors rotating at different speeds. A quantum interference mechanism of nonspecific magnetic effects that considers processes involving rotations has been previously proposed^23^ and developed in^40^ p. 266–275. This mechanism predicted that rotations affect the *H*-dependences. This *a posteriori* prediction is in agreement with experiment^17^, where a complex multi-peak *H*-dependence of a cell culture response to MF has been observed.

Essentially, it has been shown that the magnitude of HMF effect at *h* = *H* = 0 decreases for rotating MF sensors. It is easy to deduce that the characteristic feature of the HMF effect–an abrupt change in the measured value with reducing *H* to zero–is merely shifted to some other value of *H* that depends on the target rotation speed. In the *H*-dependence, this generates a kind of “window,” where the magnetic effect can be observed. The ion interference mechanism’s^23^ capability of explaining multi-peak *H*-dependences is reproduced in the LMM adapted for rotations. At the same time, the LMM allows one to determine the thermal relaxation time of the primary MF target (magnetic moments) from experimental data, which is a significant advantage.

Recently we have shown that the magnitude of a hundred different HMF effects correlate neither with the HMF value, nor with the period of the exposure to HMF, nor with their product, or “dose”^4^. MF-gradients in these HMF experiments were much smaller than those measured inside cell incubators^41^. For this reason, the wide spread of observable HMF values that cause magnetic effects cannot be explained by the uncontrolled MF variability inside MF exposure systems. At the molecular level, random MFs could be produced by paramagnetic ions like ^25^Mg^2+^ and ^67^Zn^2+^ in an uncontrolled amount^42^ and so be involved in nonspecific magnetic response. This could explain the spread of critical fields in different biological species, but not the asymmetry and the multi-peak character of the MF-dependences.

In our view, the lack of correlation strongly suggests that there is no MF sensor with the same magnetic properties for all organisms. The lack of a general MF sensor and the necessity of gene expression for nonspecific effects suggest their random nature and their link to the varying rotations that bring about the dispersal of magnetic properties of the MF sensors.

Rotations are by far not the only factor affecting the rate of biophysical events induced by the precession of magnetic moments. Many physical factors simultaneously affect the value of a biological response. “External” controllable factors or parameters include the value of a constant MF, the amplitude and frequency of the alternating MF, the angle between the constant and variable MFs, the presence of any static electric field, background variable fields, and the orientation of the MF vector with respect to the gravity vector. In addition, there will be the “internal” uncontrollable and unknown parameters including the gyromagnetic ratio of the magnetic moments involved in the magnetic response, time of thermal relaxation, the average rate of the “signal” influence of magnetic moments on the immediate molecular structure, and, as is now clear, the frequency and direction of the MF sensor rotation, i.e., the vector **Λ** in Fig. 1. To these physical factors, one adds the physiological factors responsible for the MF signal transduction from the primary physical level to an observable.

The variety of essential factors and the random character of their incoherent action make nonspecific magnetic response significantly distinct from specific magnetoreception which is dependent on specialised receptors.

## Level-Mixing Mechanism of Nonspecific Effects

We have previously proposed a physical mechanism of nonspecific response to MF in organisms^6^. This considered a nonuniform precession and thermal relaxation of a magnetic moment in the MF of parallel dc and ac components–the LMM. There are no quantum transitions caused by such a MF, and therefore it is sufficient to use the classical model of Larmor precession. Here we extend the model to the case of a rotating molecular structure that encloses one or more precessing magnetic moments.

The LMM assumes that a biological response occurs where, within the relaxation time period, the MF disturbs the dynamics of the magnetic moment so that the deviation from the state of the undisturbed uniform precession becomes significant. In the above referenced work we derive the following equation of motion in spherical co-ordinates for a precessing magnetic moment under applied dc and ac magnetic fields,

1 $$\phi (t)=\gamma Ht+\frac{\gamma h}{{\rm{\Omega }}}\,\sin \,({\rm{\Omega }}t)$$

where *φ* is the precession phase, or an azimuth angle, *γ* is the gyromagnetic ratio, *H* is the dc MF magnitude, and *h* and Ω are the amplitude and frequency of the ac MF. In the absence of the ac MF, at *h* = 0, a uniform precession takes place: *φ*(*t*) = *γHt*, where *γH* is the Larmor frequency. For a weak MF effect to cause a biological response, this background precession should be disturbed. As shown, an effective disturbance can be achieved either by a significant decrease in the dc MF or by modulating the dc field with an ac one. In the first case, the precession stops: $\dot{\phi }=0$.

Further, a concept of “reaction” was defined: it is the change in the state of the biophysical environment that immediately surrounds the precessing moment. The probability of the event to occur in the time interval [*t*−*τ*/2, *t* + *τ*/2] was assumed to be e.g^43^.

2 $$p\,(t,\,\tau )=1-\exp \,[\,-\,{\int }_{t-\tau /2}^{t+\tau /2}\,\lambda \,(u)\,{\rm{d}}u]$$

where *λ* is the density of the Poisson process and *τ* is the thermal relaxation time. If magnetic moments precess, their oscillations transform to those in the rate of downstream events *λ*. The simplest idealisation of this fact is

3 $$\lambda =\beta [1+\,\cos \,(\phi -\xi )]$$

where the proportionality factor *β* with dimension of frequency is introduced, and *ξ* denotes the random magnetic moment direction that maximises *λ*. The density is maximal when the directions *φ* and *ξ* coincide.

The basic distinction from the RPM can be surmised by examination of Eq. 3. The latter contains the phase difference between the precessing magnetic moment and a locally fixed direction *ξ*, rather than, between the phases of two precessing electron moments; i.e., the absolute rather than the relative phase is taken into account.

Based on Eqs 1–3, the probability of biophysical events *P*(*H*, *h*, Ω, *γ*, *τ*, *β*) ≡ 〈*p*〉_*t*,*ξ*_ averaged over *t* and *ξ* was derived and is dependent on six quantities. The first three of these are MF variables, and the three others are MF sensor parameters: the gyromagnetic ratio *γ* and the parameters *τ* (relaxation time) and *β* (mean rate of the biophysical events initiated by the precessing moments). The difference *P*(*H*, *h*, Ω, ...) −* P*(*H*, 0, Ω, ...) describes the probability change under ac/dc MF exposure and shows the maximum effect at *h* = 1.8*H* and Ω = *γH*, which is in agreement with many experiments; see for example p. 307–314 in^40^.

As stated in the Introduction, our aim is to extend the LMM to the case of a precessing moment located in a rotating biophysical object, Fig. 1a, under only static MF exposure, i.e. at *h* = 0. Then, under a dc MF that is decreasing from the geomagnetic field *H*_g_ to an HMF *H*, the probability change Δ*P* ≡ *P*(*H*, 0, ...) −* P*(*H*_g_, 0, ...) equals^6^

4 $${\rm{\Delta }}P(H,\,\gamma ,\,\tau ,\,\beta )\approx -\frac{1}{4}{\beta }^{2}{\tau }^{2}{{\rm{e}}}^{-\beta \tau }{{\rm{s}}{\rm{i}}{\rm{n}}{\rm{c}}}^{2}\,(\gamma H\tau /2)$$

where *P*(*H*_g_, 0, ...) is assumed to be *P*(∞, 0, ...), and sinc(*x*) ≡ (1/*x*)sin(*x*). This equation describes the HMF effect, that occurs when the dc MF is decreased from *H*_g_ to HMF *H*, see also Fig. 6 in^6^.

For convenience in what follows, the HMF effect will be associated with −Δ*P*, i.e., with a positive magnitude. It will be shown that rotations of MF sensors shift the MF at which the biological effect occurs from the range of MFs close to zero to higher MFs. We will refer to this as a static magnetic field effect. Extension of the theory presented above to a rotating MF sensor requires modification of Eq. 3.

First, besides *β*–the undisturbed rate of biophysical events generation–another parameter, the depth of modulation of *β* should be introduced. However, the value of this additional parameter would be determined by a specific biophysical mechanism indicating the type and characteristics of the MF sensor, whereas our model, being a physical one, should be formulated independently of biophysical details. Therefore, we leave this additional parameter equal to unity and note that the absolute value of the actual magnetic effects can be either larger or smaller than those calculated. Thus, the purpose of the theory below is to calculate the MF-dependencies rather than the magnitude of a magnetic effect. It is the specific form of these dependences that can be used for theory verification–by comparing it with the same type of dependence obtained through experimentation.

Second, let **m** be a unit vector in the direction of a precessing magnetic moment and **b** one in the direction that is associated with the biophysical environment. This vector is defined so that *λ* acquires a maximum value when **m** points along **b**. Then, a generalisation of Eq. 3 includes a scalar product of **m** and **b**:

5 $${\rm{\lambda }}=\beta \mathrm{(1}+{\bf{mb}})$$

**Figure 2:**
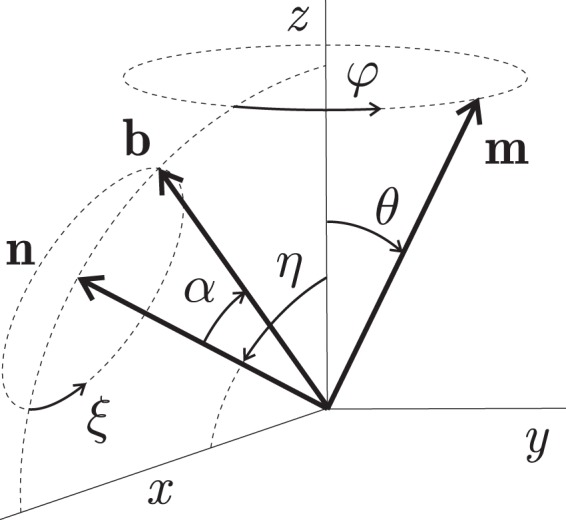
Unit vectors and their polar angles in a spherical coordinate system. Dash-line circles are the tracing of the precession of vector **m** and the rotation of vector **b** around **n**; vector **b** is shown at *t* = 0. The *z*-axis corresponds to the direction of vector **H**.

Let **n** ≡ **Λ**/Λ be the unit vector of the MF sensor rotation, and the Cartesian coordinates are chosen so that axis *z* is directed along **H** and axis *x* is in the plane formed by vectors **H** and **n**, Fig. 2. Let the target rotate about **n** with angular frequency **Λ**, which means a rotation of vector **b** so that *ξ* = Λ*t*. Note that due to subsequent time averaging and practical incommensurability of the rates of precession and rotation, their phases are not significant, and we assume the vectors **m** and **b** at *t* = 0 are in the *xz* plane. Also, for convenience of further calculations, in the definition of **b**, we use angle *α* rather than its polar angle. The angle *α* is that between **b** and **n**. Vector **m** of the magnetic moment precesses about **H** so that *φ* = *γHt*, Eq. 1.

With the above notation, one can find the Cartesian components of vectors **m** and **b**: *m*_*x*_ = sin(*θ*)cos(*γHt*), *m*_*y*_ = sin(*θ*) sin(*γHt*), *m*_*z*_ = cos(*θ*), *b*_*x*_ = cos(*α*) sin(*η*)−sin(*α*) cos(*η*) cos(Λ*t*), *b*_*y*_ = −sin(*α*) sin(Λ*t*), *b*_*z*_ = cos(*α*) cos(*η*) + sin(*α*) sin(*η*) cos(Λ*t*). Then the density of biophysical events *λ* initiated by precession can be calculated from Eq. 5. Substitution of *λ* = *λ*(*t*, *β*, *θ*, *η*, *α*, *γ*, *H*, Λ) into Eq. 2 gives a result that needs to be further averaged over time and random variables. One should keep in mind that random variable *θ* and random variables *η* and *α* are of different type with regard to averaging, for the following reasons.

The polar angle *θ* is that of the magnetic moment vector **m** at *t* = 0. The orientation of this vector should be considered random for each MF sensor separately. This means the averaging over *θ* should be performed unconditionally. The initial orientation of **m** is arbitrary in a full solid angle, hence the result generally must be averaged over the azimuth and polar angles. However, the result does not depend on the azimuth angle, and only the unconditional averaging over *θ* remains.

In contrast, the positions of the rotation vector **n** and target vector **b** at *t* = 0 that are given by angles *η* and *α* respectively, Fig. 2, have definite values for each specific target. Averaging over these angles makes sense only if they have variable random values in different targets. Note that this is not the case if the targets of the same type have a predominant orientation. For example, many plant cells are oriented in a certain way relative to the gravity vector. Consequently, rotations of macromolecules carrying MF sensors could inherit this preferred orientation in the form of a more or less definite orientation of the vector **n** and vector **b** at *t* = 0. Thus, it is relevant to study two cases, (i) deterministic and (ii) stochastic with uniformly distributed random values of *η* and *α*.

Substituting Eq. 5 in Eq. 2 and performing averaging over time and *θ*, one can derive the probability of the secondary events initiated by a precessing magnetic moment, i.e., *P* = *P*(*H*, *γ*, *τ*, *β*, Λ, *η*, *α*),

$$P=\frac{1}{\pi T}{\int }_{0}^{\pi }{\int }_{0}^{T}[1-{\rm{e}}{\rm{x}}{\rm{p}}(\,-\,{\int }_{t-\tau /2}^{t+\tau /2}\lambda \,{\rm{d}}u)]{\rm{d}}t\,\sin (\theta ){\rm{d}}\theta $$

where *T* ≡ 2*π*/|*γH*−Λ| is the period of a two-frequency oscillating process. Finally, we arrive at an expression for the probability change that is suitable for comparison with experiment,

6 $${\rm{\Delta }}P(H,\,\gamma ,\,\tau ,\,\beta ,\,{\rm{\Lambda }},\,\eta ,\,\alpha )\equiv P(H,\,\gamma ,\,\mathrm{...})-P(\infty ,\,\gamma ,\,\mathrm{...})$$

As is seen, three new variables are added to Δ*P* as compared with the case of fixed MF sensors. As follows from Eqs. 4 and 6, these variables are the speed Λ of the MF sensor rotation and the angles that define axis of rotation **n** and vector **b** of the rotating sensor.

**Figure 3:**
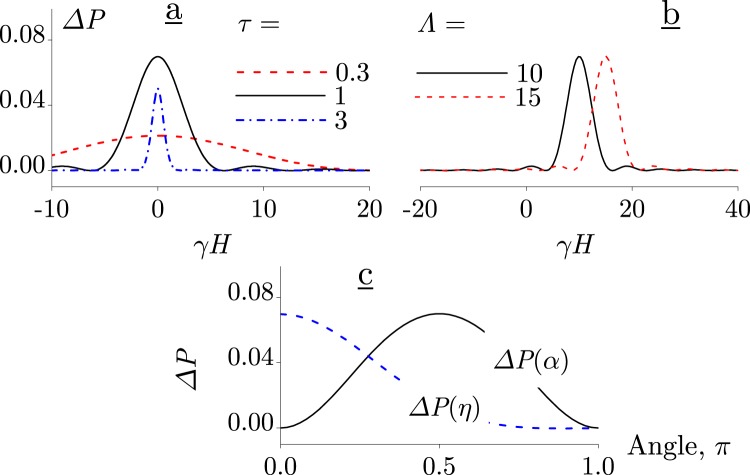
Static MF effect in different modes at *β* = 2. Dependences on *γH* at *η* = 0 and *α* = *π*/2: (**a**) a resonance-like response, Λ = 0; (**b**) the asymmetry of the response regarding a MF reversal, Λ = 10 and 15, *τ* = 1. (**c**) Dependences at *τ* = 1, *γH* = 10, and Λ = 10: on *α* with *η* = 0 and on *η* with *α* = *π*/2.

As an analytical evaluation of Eq. 6 would be too cumbersome, a few different cases have been studied numerically and are presented in Fig. 3.

If **Λ** = 0 or the vector **b** is parallel to **n**, i.e., *α* = 0, this obviously reduces to the case without rotation. The half-width of peak at *H* = 0 is consistent with Eq. 4: half-width is defined by the argument of the cardinal sine function where it rapidly changes, i.e., $\Delta H \sim \mathrm{1/(}\gamma \tau )$.

In the case of rotation, the most pronounced result occurs where the axis of rotation coincides with the *z*-axis and the MF sensor vector **b** is perpendicular to the *z*-axis, that is *η* = 0 and *α* = *π*/2. Then, the probability of biophysical events that are caused by precessing magnetic moments has a resonance-like peak, Fig. 3b, provided the angular velocity of rotation and the MF vector are linearly related, ${\boldsymbol{\Lambda }} \sim -\,\gamma {\bf{H}}$. As is seen the position of the peak shifts in proportion to Λ. That is the level mixing, or a slow precession, occurs at values $H \sim \Lambda /\gamma$. To improve clarity, this behaviour can also be described in another way. In the laboratory system of reference, the frequency of precession *ω* = *γH* is proportionate to MF *H*. As *H* is ramped, the frequency of precession is altered which “probes” the immediate rotating environment with different frequencies. When the frequency coincides with that of the environment rotation (the sensor rotation), i.e., *ω* = Λ, a response appears. Hence this introduces the concept of *H*-dependent spectroscopy for rotating MF sensors.

The position of the peak Fig. 3b is independent of the values of all variables other than Λ. This enables one to study the *η*- and *α*-dependences. While the effect is in its maximum, one can examine how it depends on the orientation of the axis of rotation **n** and that of the sensor vector **b**. As seen in Fig. 3c, these dependences are rather smooth. This creates an opportunity to observe the static MF effect even for arbitrary or unknown values of these angles.

As indicated above, the rotation axes of macromolecules can inherit a preferred orientation of cells; then the values of *η* and *α* can be considered definite. This case corresponds to the results shown in Fig. 3b. How will these results change if the values *η* and *α* are random rather than definite? Let both angles, being polar angles, be distributed in the range [0, *π*). Since the position of the peak in Δ*P*(*γH*) does not depend on these variables, it is sufficient to average the magnitude of the effect over these angles only in the centre of the peak.

The result shows that the peak height decreases by more than an order of magnitude, from 7% to about 0.5%. The latter is an order of magnitude larger than the RPM effect observed in MFs similar to the geomagnetic field (see Appendix). However, this value is, nevertheless, small for a reliable explanation of the biological effects of static MF. This suggests that for a static MF effect to occur, some kind of rotation ordering is desirable. However it is not necessary for non-rotating MF sensors; they show a 14% HMF effect (as follows from Eq. 4 at *τβ* = 2) independent of molecular rotations.

## Discussion

Magnetic nonspecific effects occur where MFs effectively combine with other physico-chemical and physiological factors. These latter factors are difficult to control, and the occurrence of magnetic effects is, on the whole, difficult to predict. However, these effects occur more often under the following conditions^4^,

7 $${\rm{\Omega }}/\gamma H\sim 1,\,\,\,h/H\sim 1,\,\,\,\gamma H\tau \lesssim 1$$

of which the first two approximate equalities relate to the biological effects of the ac/dc MF with amplitude *h* and frequency Ω of the alternating MF component, and the last inequality to the HMF effects.

As has been shown above, it is reasonable to assume that the MF sensors, at least a significant portion of these, reside on rotating macromolecules or related enzymes. Biophysical MF sensors that rotate have, in addition to *γ* and *τ*, another parameter which is the speed of rotation **Λ**. Addressing the sensor rotations significantly changes the basic relationships in magnetobiology. For sensors carrying moments with a gyromagnetic factor *γ*, their rotation with angular velocity ${\boldsymbol{\Lambda }}||{\bf{H}}$ is equivalent to the presence of an additional MF **H** = −**Λ**/*γ*. Accordingly, in this exemplar case, Eq. 7 can be generalised as follows,

8 $${\rm{\Omega }}/|\gamma H-{\rm{\Lambda }}|\sim 1,\,\,\,\,h/|H-{\rm{\Lambda }}/\gamma |\sim 1,\,\,\,\,|\gamma H-{\rm{\Lambda }}|\tau \lesssim 1$$

At Λ = 0, these relations are reduced to those shown in Eq. 7. The latter of Eq. 8 is for the static MF effects, and it clearly demonstrates asymmetry of the magnetic response with regard to a MF reversal.

Thus, in terms of the LMM, when measuring the MF biological effect in different modes of the magnetic exposure and rotation of the samples, one can determine not only the gyromagnetic factor and thermal relaxation time of the magnetic moments of an MF sensor, but also the speed of its rotation. Such a set of parameters would make it possible to identify the MF molecular sensors with greater reliability. That the LMM can extract information on molecular rotations from experimental data makes the research in HMF and static MF effects almost the only effective method for finding MF sensors, i.e., magnetosensitive macromolecules that were not selected for magnetoreception during their phylogeny.

The MF-dependence as seen in Fig. 3b is not symmetric with respect to a MF reversal, which can be qualitatively compared with currently available experimental data and the outcomes of suggested future experiments. Qualitative testing is important because its conclusions do not depend on the nature of magnetic sensors, but depend on the theory from which the conclusions are derived. In this way, the LMM can be verified.

Regarding the available data, the gene expression in *A. thaliana*^7^ and calpain activity in mollusk *L. stagnalis*^8^ are not symmetrical with respect to the static MF reversal. Many migrating animals demonstrate an asymmetric magnetic response^9,10^ known as the “polarity compass” whose nature remains unclear. The LMM–in its “rotation” extension–could explain such “polarity compass” results. Note that the “inclination compass” that is also observed in magnetic navigation in animals^44^ and is insensitive to the MF “polarity” is explained, most likely, by the RPM^3^.

Regarding future experiments, the LMM can be directly tested because it predicts that (a) in plants that have their magnetically responsive cells predominantly aligned in parallel with the gravity vector, the biological response to MF will be *asymmetric* when the magnetic field vector is reversed with respect to the direction defined by the gravity vector, and conjunctively (b) the magnetic response will be *symmetric* at the reversal of the magnetic field vector lying in the plane perpendicular to the gravity vector. MF reversing should not be rapid, in order to avoid any confounding contribution of the induced electric currents.

Note that a qualitative feature of Fig. 3b is the spectral peak, and many experiments^13,17,18^ confirm the spectral character of *H*-dependences. Thus, two general features that have been observed experimentally are at the same time a hallmark of the LMM: (i) the absence of symmetry and (ii) the presence of spectral peaks.

A quantitative comparison may not be as crucial as a qualitative one, but enables the building of grounded hypotheses on the nature of magnetic sensors. Although there are more than two hundred articles, documenting nonspecific HMF effects in organisms^4^, these data have not, in general, investigated detailed MF-dependences needed for quantitative comparison to the predictions of the LMM. However, recently, a set of MF stimulus-response curves for gene expression has been obtained in a study of plant germination with MFs ranging from about 0.5 to 188 μT^13^, allowing a preliminary comparison between theory and experiment. In this work, the *H*-dependence of the relative transcript amount of *rbcl* (large subunit of ribulose bisphosphate carboxylase/oxygenase) in seedlings of *A. thaliana* raised for 120 h under broad-band blue light showed two spectral peaks.

The properties of a spectral peak can be used for identifying the magnetic moments involved in nonspecific magnetic effects and the speed of rotation of their carrier. The first peak in the above mentioned experiments^13^ was centred at 50 *μ*T with a half-width of 10 *μ*T. The last of Eq. 8 predicts the peak centre *Hp* and its half-width Δ*H* to be

9 $${H}_{{\rm{p}}}\sim {\rm{\Lambda }}/\gamma ,\,\,\,{\rm{\Delta }}H\sim 1/(\gamma \tau )$$

These two equations bind three unknown quantities: the gyromagnetic ratio *γ* and the thermal relaxation time *τ* of the precessing magnetic moment located at the sensor rotating with angular speed Λ. The choice of *γ* is limited to that of spinning or orbiting electrons, protons or magnetic isotopes, and molecular gyroscopes^20^. Then, *γ* can be substituted into Eq. 9, and Λ and *τ* can be calculated at the indicated empirical values of *H*_*p*_ and Δ*H*. In this way, one finds that sensors, based on the precession of electrons, protons, and amino-acid residues in proteins, must rotate with speeds on the order of a MHz, KHz, and a few Hz, respectively; with these speeds, the thermal relaxation time must be, correspondingly, 0.5 *μ*s, 0.5 ms, and 0.1 s. In our view, a proton is the most likely primary target in the magnetic effects^13^ observed in plants.

The reliability of this preliminary identification might benefit from more detailed and generalised experimental data. Probably, the theory should take into account the often intermittent character of molecular rotations, like in RNA and ATPase. In addition, Ω-dependencies obtained in the same organism under the ac/dc MF exposure, as explained in^4^, could provide direct information on the gyromagnetic ratio of the primary targets.

Studies on the HMF effects are important for future space flights that are featured by MFs more than a thousand-fold smaller than the geomagnetic field. For this reason, when studying the magnetic effects on Earth, researchers model the space conditions by correcting for gravity. This is usually achieved with clinostats that rotate samples so that the gravity vector in the frame of the sample is averaged to zero. Due to the influence of rotations on the nonspecific magnetic effects, the accepted interpretation of the results obtained in clinostats should be revised.

The LMM is a general physical mechanism that explains nonspecific response to MF regardless of the biophysical construct that hosts a precessing magnetic moment. The biophysical construct should be taken into account in less abstract models that would be aimed at tracing the propagation of magnetic signal at each level, i.e., in models that incorporate the coupling of the initial detection of the magnetic field (detection that is described by the LMM) with the subsequent biophysical/biological train of events that result in an observable. Examination of Fig. 1, for example, raises the issue of what might be the biophysical object whose state changes coherently with the state of a precessing magnetic moment. Modeling this will require a separate study.

In summary: (i) the Level Mixing Mechanism as extended to the case of molecular rotations identifies primary physical causes for, and thus explains key features of the observed MF-dependences including asymmetry with regard to the static MF reversal and resonance-like peaks, (ii) the LMM predicts easily verifiable symmetry relations of magnetic effects in some plants, (iii) molecular rotations are a significant factor affecting nonspecific magnetic effects in organisms, and (iv) fundamental biological information on the molecular rotations can be obtained from the shift of the spectral peaks. The non-invasive extraction of such information on the rotation of sub-cellular structures introduces the potential to use LMM as the basis for a new biological spectroscopy.

## Appendix. Radical Pair Mechanism

Is it feasible that the multi-peak structure of *H*-dependences, like that in^13,17,18^, could also be explained on the basis of the RPM^3^, thus presenting the sum of the dependences of different genes, each one having its specific position of the extremum?

In principle, every type of radical pair taken with its immediate environment has its own position of the minima in the *H*-dependence of the RPM product yield–the so-called low-field effect, or LFE^45^. This means that the simultaneous presence of such MF sensors could, in principle, form an *H*-dependence with a few peaks or wells. However, there are problems that make this scenario unlikely for nonspecific magnetic effects in MFs like the geomagnetic field^4^.

Problem one: For the RPM, there are a few interdependent physicochemical times that should be in certain relations to each other for the LFE to be achieved. Most limiting is that the chemical lifetime of the radical pair should be on the order of the time of spin coherent motion that is limited by the electron thermal relaxation time, usually to 10^−9^–10^−7^ s. This decreases the chances to observe the effect.

Problem two: The MFs that maximise LFE in experiments are usually about 1 mT. This is two to three orders of magnitude greater than the HMFs that cause a variety of nonspecific magnetic effects^4^. Moreover, the LFE peak in *H*-dependence is wide, and in order to resolve even two peaks one would have to scan the MF interval of at least 4–6 mT whereas the work^13^ reported a few peaks within the narrow interval of 100 *μ*T.

Problem three: The LFE magnitudes that have been observed experimentally reach at most 0.1% with MF switching between zero and the geomagnetic field magnitude. As the first problem, this one originates also from the small thermal relaxation time of the radical pair electrons. Sometimes, researchers speculate that this time could be a hundred or even more times greater than 10^−7^ s, thus providing approximately proportional growth in the effect. However, there is still no direct laboratory confirmation. LFE magnitudes could be amplified due to the chemical bifurcation phenomena^46^, however this qualitative hypothesis does not imply experimental verification.

These problems may have been overcome to allow the RPM to explain bird detection of the geomagnetic fields due to special anatomical adaptations in the birds eye that have not been realised with respect to nonspecific magnetic field effects. Cryptochromes that carry radical pairs are arranged, as often suggested^3^, orderly so that flavine-tryptophan radical pairs transduce their signals coherently each to about one nerve from millions in the eye. In this case, a statistical enhancement of the signal-to-noise ratio is possible and the bird’s brain gains 100 and more amplification of the magnetic sensitivity, which is enough to explain magnetic orientation and navigation in animals. This could be considered a particular case of the trans-disciplinary mechanism, metaphorically called “Many Wrongs Principle”^47^. It involves the central limit theorem of mathematical statistics in order to explain amplification of biological sensitivity to various factors in a group of individual sensing elements and even in a group of animals^48^. Plants, however, do not have such a specific amplification mechanism. So there are three reasons–mT-range, low peak resolution, and a small relative magnitude–that make the RPM unlikely as a primary mechanism explaining nonspecific magnetic effects in plants, particularly the multi-peak *H*-dependence of gene transcript^13^. Moreover, a fourth problem exists–it is the symmetry of the RPM with regard to static MF reversal, which is not consistent with the above cited experiments.

The RPM symmetry regarding MF reversal **H **↔ −**H** can be illustrated by the following simplified consideration. The core of the RPM is singlet-triplet transitions. In the canonical case of two noninteracting electrons, one of which is in the hyperfine MF **H**_0_ of its proton, the singlet-triplet transitions are caused by the modulation of the hyperfine interaction. Roughly, the singlet-triplet transitions occur due to the modulation of the hyperfine field by a much weaker additional MF **H**_1_. Of the two components of this additional field–a parallel and a perpendicular to **H**_0_–only the latter one denoted **H** causes quantum transitions of that electron and hence the singlet-triplet conversion of S → T_±_ type that is often associated with LFE. However, the direction of this component is arbitrary in the plane perpendicular to **H**_0_. This means that the probability of a singlet-triplet conversion can depend only on the absolute value of **H**.

In the Heisenberg picture, the electron spin wave function *ψ* at time *t* satisfies equation

$$|\psi (t)\rangle =\exp \,[i\gamma t{\boldsymbol{\sigma }}({{\bf{H}}}_{0}+{\bf{H}}\mathrm{)/2]}|\psi \mathrm{(0)}\rangle $$

where *γ* is the gyromagnetic ratio, and ***σ*** are the Pauli matrices. Let the initial state |*ψ*(0)〉 be an eigenvector *ψ*_0_, and |*ψ*(*t*)〉 be superposition *c*_0_(*t*)*ψ*_0_ + *c*_1_(*t*)*ψ*_1_. Then a straightforward calculation gives the probability ${c}_{1}^{\ast }{c}_{1}$ of the spin to occur in the state *ψ*_1_ at time *t* as $w={\sin }^{2}(\gamma Ht\mathrm{/2)}$. This function corresponds to the decrease in the probability of the initial singlet state of the radical pair, and the increase in the probability of the triplet T_±_ states. A chemical product yield depends on the MF only through *w* and, hence, is symmetric regarding the MF reversal.

Keeping in mind the linearity of the quantum equations that describe the evolution of the spin states and of the respective amounts of radical pairs, one can estimate the relative product yield as averaged *w*. By averaging $w\leftarrow (1/\tau )\,{\int }_{0}^{{\rm{\infty }}}\,w(t)\exp (\,-\,t/\tau )\,{\rm{d}}t$ over the interval of the thermal relaxation time *τ* and averaging $w\leftarrow (1/{\rm{\Omega }})\,{\int }_{{\rm{\Omega }}}\,w\,{\rm{d}}{\rm{\Omega }}$ over the full solid angle Ω for the random direction of vector **H**_1_ with respect to **H**_0_, we obtain the result as an approximate formulation, the absolute accuracy of which versus the lengthy exact solution is better than about 0.02,

10 $$\frac{1}{2}[1-\frac{1}{1+\frac{1}{2}(\gamma H\tau {)}^{2}}]$$

This function is close to one derived in^45^ for the maximum theoretical limit of the relative LFE product yield,

$$\frac{2}{5}[1-\frac{1}{1+{(\frac{1}{2}\gamma H\tau )}^{2}}]$$

where we have replaced *τ*_chem_ by *τ*, while implying that the thermal relaxation time is smaller than the chemical lifetime and, thus, is a bottleneck of the spin evolution that defines the LFE magnitude. Literally, this latter function is not shown in^45^, however this is equivalent to their Eq. 54. Thus, the relative product yield, being a quadratic function of *H*, is symmetric to the MF reversal.

Indicative data on the RPM sensitivity to MFs based on the values of the LFE can be obtained. Within an order of magnitude and at the time intervals of practical interest, theoretical sensitivity of the reaction yield to the MF is nearly proportionate to the chemical lifetime *τ*_chem_ of radical pairs. This proportionality has been shown in many publications in the approximation of absence of thermal relaxation. Then, a characteristic of sensitivity is its relative value *s* determined as the effect magnitude per unit MF and per unit of the chemical lifetime (magnitude in percent divided by *H* and *τ*_chem_). Since the time of a coherent radical pair evolution, i.e., when the reaction is magnetically sensitive, is limited by the thermal relaxation time, one can evaluate the corrected theoretical sensitivity to MF as *S* = *sτ*, where *τ* ~ 100 ns is the highest thermal relaxation time, Table 1. There are many other publications that contain data on the LFE, however they would not change the pattern already seen in the Table. Even the optimistic computational and greatest experimental sensitivities, 0.05 and 0.001% per *μ*T respectively, are orders of magnitude less than those experimentally observed in most of nonspecific magnetic effects in organisms^4^. Other criticism of the RPM mechanism as applied to animal magnetic sense can be found in^49^.

**Table 1:** Theoretical and experimental sensitivity ^§^ of the RPM to MFs.

Effect, %	MF, *μ*T	*τ*_chem_, ns	*s*, % per *μ*T ×ns	*S*, % per *μ*T	Ref.
20	50	227	1.8 × 10^−3^	0.18	^45^^−^
16	50			0.16	Eq. 10^−#^
5	100	100	5 × 10^−4^	0.05	^51^*
18	100	1000	1.8 × 10^−4^	0.018	^52^*
4	100	1000	4 × 10^−5^	0.004	^2^*
6.7	50	5000	2.7 × 10^−5^	0.0027	^3^*
1	1000			10^−3^	^53^^+^
1	2000			5 × 10^−4^	^54^^+^

^§^*S* values are order of magnitude estimates, ^−^rough analytical limits, *more realistic computational estimates, ^+^experimental *in vitro* studies, ^#^*τ* = 100 ns.

A photogenerated radical pair (not necessarily magnetosensitive) could, nevertheless, be somehow involved as a secondary agent, after LMM generates a signal. The transduction of this primary magnetic signal to the biological level would then be modified by a photofluence depending on its spectrum.
